# First Survey of the Vascular and Cryptogam Flora on Bulgaria’s Ancient Mounds

**DOI:** 10.3390/plants11050705

**Published:** 2022-03-06

**Authors:** Iva Apostolova, Desislava Sopotlieva, Magdalena Valcheva, Anna Ganeva, Veselin Shivarov, Nikolay Velev, Kiril Vassilev, Tsvetelina Terziyska, Georgi Nekhrizov

**Affiliations:** 1 Institute of Biodiversity and Ecosystem Research, Bulgarian Academy of Sciences, 2 Gagarin Str., 1113 Sofia, Bulgaria; dsopotlieva@gmail.com (D.S.); magdalena.i.valcheva@gmail.com (M.V.); annaganeva8@gmail.com (A.G.); v.shivarov@abv.bg (V.S.); nikolay.velev@abv.bg (N.V.); kiril5914@abv.bg (K.V.); ts.terziyska@gmail.com (T.T.); 2 National Archaeological Institute with Museum, Bulgarian Academy of Sciences, 2 Saborna Str., 1000 Sofia, Bulgaria; nehrizov@gmail.com

**Keywords:** bryophytes, generalist plants, grassland specialists, historical monuments, invasive alien plants, kurgans, lichens, native plants

## Abstract

This work represents the first study of the floristic diversity on Bulgaria’s ancient mounds. The objective of this research was to assess the importance of the mounds for the preservation of the native vascular and cryptogam flora. Our sampling design included 111 ancient mounds distributed throughout the country. We recorded a total of 1059 vascular plants, 58 bryophytes and 61 lichen taxa. Despite their small area, the mounds were shown to preserve nearly a quarter of the Bulgarian flora. The vegetation cover on the mounds included 61% perennials indicating a long-term persistence and stability. The majority (98%) of the established vascular plants were native species. Although the conservation significance of the vascular plant species were not common, we recorded 2 critically endangered, 9 endangered and 14 Balkan endemics during the present study. The lichen *Arthopyrenia salicis* was recorded for the first time in Bulgaria and a new locality of the rare bryophyte *Ceratodon conicus* was discovered. The established compositional difference between plots from the northern and southern slopes of the mounds (88.95%) is a testament to the high local habitat diversity. The prevalence of species characteristic for *Festuco-Brometea* suggests that the mounds preserve fragments of native grasslands and steppes. The variation in cover of agricultural and other human modified areas in the mounds’ immediate surroundings did not substantially affect their species richness. We argue that the ancient mounds should be taken into consideration in future green space planning.

## 1. Introduction

Ancient mounds (also called tumuli or more commonly kurgans) were constructed in temperate Eurasia between 4th millennium BC and 4th century AD and used primarily for burial purposes. A remarkable number of these mounds have been preserved due to their spiritual and cultural importance [1,2,3,4]. Bulgaria is exceptionally rich in ancient mounds with a known number of approximately 50,000 [5]; 11,000 of these mounds have been registered in the Archaeological Map of Bulgaria (http://www.naim-bas.com/akb/ accessed on 3 November 2021). Some of Bulgaria’s ancient mounds are remarkable historical monuments, including massive underground stone buildings often decorated with wall paintings (e.g., Kazanlak and Aleksandrovo tombs). The most attractive of these structures are important tourist destinations open to the public. At present, most of Bulgaria’s ancient mounds are surrounded by vast agricultural lands [4]. Similar to ancient mounds in other European countries, these structures are often standing as sole “islands” of semi-natural vegetation in an otherwise human-modified landscape [3,6,7]. Along with field margins, road verges and buffer strips adjacent to arable land, the ancient mounds preserve small semi-natural fragments and provide an opportunity for the long-term survival of indigenous flora. Moreover, ancient mounds consist of different microhabitats, which enrich the suitability for the development of an ecologically diverse flora [8,9]. The long-term persistence of the mounds within agricultural lands, primarily due to sacred and religious respect, naturally makes them a part of the Green and Blue Infrastructure defined at the European level as a “strategically planned network of natural and semi-natural areas with other environmental features designed and managed to deliver a wide range of ecosystem services” [10].

Recently, there has been an increased interest in burial mounds as biodiversity hotspots situated in an otherwise homogenous agricultural landscape [2,3,7,11,12,13,14,15]. Plants, and especially flowering plants (Angiosperms), are one of four groups of living organisms (along with Heteroptera, Symphyta and aculeate Hymenoptera) that have been shown to be best served for the biodiversity evaluation of cultivated areas [16]. Recent research has shown that ancient mounds preserve a remarkable plant diversity [3,11,13,15,17,18]. To date, no studies on the natural value of Bulgaria’s ancient mounds have been conducted and no records of their floristic diversity are known to exist. At the beginning of the current research, we assumed that the cultural significance and principal sacrosanct nature of Bulgaria’s ancient mounds, akin to other countries, provided long-term repository conditions for natural communities and that they served as refugia for indigenous flora in anthropogenically transformed areas. Considering cryptograms’ signal for increased degree of community stability and naturalness [19], and in order to enrich the current biodiversity assessment, we included cryptograms (bryophytes and lichens) along with vascular plants in this survey.

The objectives of this study were (1) to collect completely novel information about the floristic diversity of Bulgarian ancient mounds and (2) to assess the potential of ancient mounds to preserve native vascular and cryptogam flora, despite being largely isolated.

## 2. Materials and Methods

### 2.1. Study Objects and Study Area

**Figure 1:**
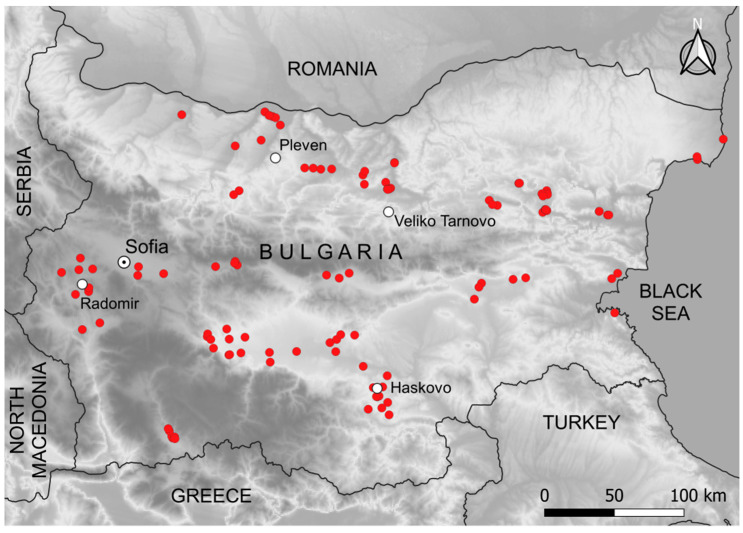
Map of Bulgaria with marked locations of the sampled mounds.

**Figure 2:**
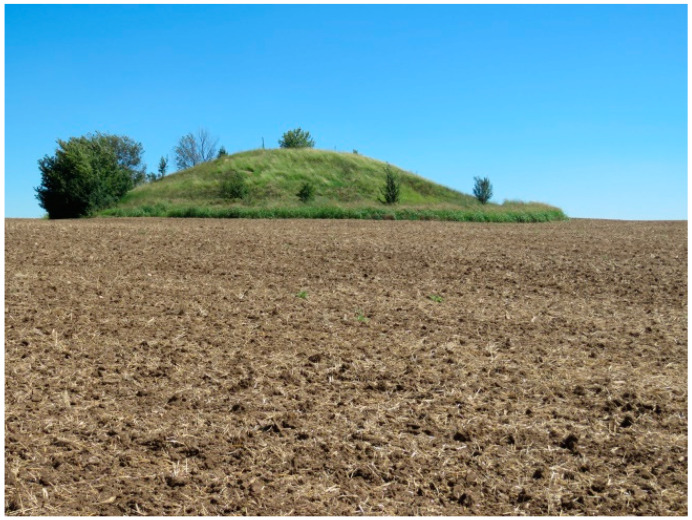
An example of a typical mound view (photo credit: I. Apostolova).

We used the Archaeological Map of Bulgaria (http://www.naim-bas.com/akb/ accessed on 3 December 2021) to select the study objects. Our selection criteria included mounds that were (1) undisturbed by archaeological investigation, (2) were clearly recognizable, and (3) were higher than 1 m and more than 9 m in diameter (Table 1). We visited a total of 111 ancient mounds spread out across the territory of Bulgaria (Figure 1 and Figure 2). The mounds were located in the lowlands and hilly plains of the country between 60 and 900 m a.s.l. In our study, a larger mound base usually corresponded to a larger height. The correlation of height to diameter was *r* = 0.57518 (*p* < 0.05) and the correlation of height to 2D area (calculated as *πd*^2^/4, where “*d*” is diameter) was *r* = 0.61815 (*p* < 0.05). Therefore, we used the 2D area of the mounds as a representation of the relative mound size.

We visually estimated the percentage of grassland and woody vegetation cover of the mounds using Google Earth images. We chose a 10% threshold to facilitate the rough estimation in vegetation cover of the mounds. There were 86 mounds with more than 70% herbaceous cover, 16 with more than 70% forest vegetation cover and 9 with mixed vegetation cover.

In order to estimate the degree of mound isolation, we created a buffer area with a radius of 200 m around the base of each mound and calculated the land cover of natural vs. non-natural habitats within. The land cover types in the buffer area were obtained from the Land Parcel Identification System (LPIS) database maintained by the Ministry of Agriculture, Food and Forestry of Bulgaria and generalized as semi-natural vegetation and agricultural and other anthropogenic lands (for details see [4]). More than half of the studied mounds were highly isolated—80 mounds were surrounded by more than 70% of agricultural and other anthropogenic lands.

The investigated mounds fall within the temperate and the continental–Mediterranean climatic zones. The temperate zone, which incorporates the northern parts of the country, has an average mean annual temperature of 11.9 °C and annual precipitation of 573 mm (town of Pleven, 1971–2000), while the continental–Mediterranean zone, which is more typical for the southern part of the country, has an average mean annual temperature of 12 °C and annual precipitation of 637 mm (town of Haskovo, 1971–2000) [20]. A major part of the study area falls within the broadleaved deciduous forests zone (Map of Natural Vegetation of Europe, [21]), while the southern areas include Mediterranean vegetation fragments with typical plant species [22]. Some small areas in the north-east are influenced by steppe vegetation [23].

### 2.2. Sampling Design, Data Collection and Data Analysis

The field work was conducted during the maximum period for vegetation development (June and July) in 2019 and 2020. Our sampling design was focused on the major ecological differences exhibited at the northern and southern slopes of the mounds. This design considered findings reported by previous studies on burial mounds [9] regarding the difference in floristic composition at different exposures due to habitat heterogeneity. In order to obtain more detailed floristic data, we first sampled all species within 5 × 5 m plots situated on the northern and southern slopes (two plots per mound). We then carefully explored the remaining mound area and recorded any additional species until the floristic variety was exhausted. We used presence/absence species data, both at plot and at mound level, for our analyses.

Despite the fact that some taxa were identified to subspecies, we set the final plant list to species level. Some closely related species were joined in species aggregates (*Achillea millefolium* aggr.–including *A. millefolium*, *A. pannonica* and *A. setacea*) or determined at aggregate level (*Rubus hirtus* aggr.). Plants that were in a phenological stage unsuitable for correct species determination, or that were difficult to identify (e.g., *Taraxacum* spp.), were determined to genus level. The vascular plant species nomenclature follows The Euro+Med PlantBase [24], with the exception of *Brassica juncea*, which follows the Plant List [25]. Bryophyte nomenclature follows Hill et al. [26], and lichen nomenclature follows Nimis et al. [27].

For each taxon, we attributed a set of characteristics regarding biological type, functional role, floristic element, conservation and native status (Supplementary ESM S1). Data regarding the biological type of vascular plants were extracted from national literature sources [28,29]. The biological types were grouped as follows: short lived (including annual and biannual plants), perennial (including biannual to perennial and perennial plants), dwarf-shrub, shrub and tree. The association of vascular plant species to higher rank syntaxa was defined following Mucina et al. [30]. In cases when more than a single phytosociological class was proposed for a certain diagnostic species, we selected the best representative for the country’s vegetation based on our expertise. The diagnostic role of a species was used to assign each species to one of the following 3 functional groups: generalists, grassland specialists and forest specialists. We considered species to be generalists if they were diagnostic of synanthropic vegetation or if they had a broad distribution across different habitat types. The functional affiliation of species that were not assigned to a specific syntaxon was determined based on their most common habitat occurrence in the country. The determination of phytogeographical (floristic) elements for vascular plants follows Assyov and Petrova [31], for bryophytes—Ganeva and Düll [32] and for lichens—Wirth [33] and Nimis [34].

The native status of vascular plants was retrieved from Euro+Med PlantBase [24] because of the lack of such data for Bulgaria. Only species listed by Petrova et al. [35] were recognized as invasive alien plants. Vascular plants with conservation importance included Balkan and Bulgarian endemics ([31,36] complemented by The Euro+Med PlantBase [24]), Bulgarian red list species [37], species protected by the Bulgarian legislation (Appendix 3 of the Bulgarian Biological Diversity Act [38]) and other European and international documents (e.g., Council Directive 92/43/EEC [39], as well as the Convention on International Trade in Endangered Species of Wild Fauna and Flora (CITES) [40]). The above mentioned attributes were not applicable for the taxa determined to genus level (N/A). The records of these species were not included in the analyses based on functional groups.

We calculated basic descriptive statistics for all biological characteristics of the registered vascular plants. We used similarity percentages analysis (SIMPER) [41] in PRIMER 7 [42] to determine the species that contributed the most to the floristic resemblances between mounds. The difference in species composition between the mounds was assessed by using the beta diversity index in PAST [43]. We used correlation analyses with Pearson correlation coefficient in STATISTICA 13 [44] to test for correlation between floristic richness (total vascular plant richness, richness of generalists and of grassland and forest specialists) and two other variables: 2D area and proportion of anthropogenic land in the buffer areas. We used plot level data to graphically express the differences in species richness of the different species groups between plots with northern and plots with southern exposure (by their mean values and standard deviation), also carried out in STATISTICA 13 [44].

## 3. Results

### 3.1. Diversity and Species Characteristics

#### 3.1.1. Vascular Plants

The list of registered vascular plants includes 1059 taxa (Supplementary ESM S1). The average number of species per mound was 69.9 ± 22.6 SD (min 27, max 152). We identified 971 plants to species level, accepted 3 taxa as aggregates or species groups, and identified 85 taxa to genus level. The floristic diversity was confined to 82 vascular plant families. Flowering plants (Angiosperms) made up the majority of the observed species diversity and only seven species belonged to other groups: one horsetail—*Equisetum hyemale*, two ferns—*Polystichum aculeatum*, *Pteridium aquilinum*, and four Gymnosperms—*Juniperus communis*, *J. oxycedrus*, *Pinus nigra*, *P. sylvestris*. There were 23 families represented by more than 10 taxa, the most species rich of these were Asteraceae—126 taxa (11.9% of the established taxa), Fabaceae—111 taxa (10.5%), Poaceae—106 taxa (10%), Lamiaceae—61 taxa (5.8%), Brassicaceae—58 taxa (5.5%), Caryophyllaceae—57 taxa (5.4%), Rosaceae—48 taxa (4.5%), Apiaceae—46 taxa (4.3%), Boraginaceae—31 taxa (2.9%) and Plantaginaceae—31 taxa (2.9%). Twenty-three other families (28.1%) were represented by a single species. The flora of the studied mounds was composed primarily of perennial herbaceous plants (61%), followed by short-lived plants, and a low number of shrubs and trees. Generalists and grassland specialists dominated the species composition of the mounds. A major part (98%) of the established vascular plants consisted of native species (Table 2). Only 21 plants belonged to other categories (alien (status unknown)—13, naturalized alien—5, in large-scale cultivation—2 and doubtfully native—1). Invasive alien plants included *Acer negundo*, *Ailanthus altissima*, *Amaranthus albus*, *Conyza canadensis*, *Cuscuta campestris*, *Datura stramonium*, *Erigeron annuus*, *Phytolacca americana*, *Robinia pseudoacacia*, *Sorghum halepense*, *Xanthium orientale* subsp. *italicum* and *X. strumarium*. These taxa represent 20% of all plants included in the list of invasive or potentially invasive alien plants in Bulgaria and three of them (*Acer negundo*, *Ailanthus altissima* and *Robinia pseudoacacia*) are among the “top 10” invasive alien plants in Bulgaria. The largest number of invasive plant species we registered on a single mound was four. The mound (ID 586) was situated in the central part of north Bulgaria (north of the town of Veliko Tarnovo). A substantial number of the studied mounds (40 or 35.4%) had at least one invasive alien plant.

Critically endangered species present on the mounds included *Anchusa stylosa* and *Limonium asterotrichum*. Endangered plants present on the mounds included *Astragalus haarbachii*, *A*. *wilmottianus*, *Chamaecytisus frivaldszkyanus*, *C*. *kovacevii*, *Dianthus pallidiflorus*, *Erysimum cheiranthoides*, *Festuca thracica*, *Goniolimon besseranum* and *Jurinea ledebourii*. Fourteen of the registered taxa were Balkan endemics: *Achillea clypeolata*, *A*. *pseudopectinata*, *Armeria rumelica*, *Astragalus wilmottianus*, *Asyneuma anthericoides*, *Cytisus eriocarpus*, *Dianthus moesiacus*, *Festuca thracica*, *Heptaptera triquetra*, *Koeleria simonkaii*, *Dichoropetalum vittijugum*, *Polygala supina* subsp. *rhodopea* (syn. *P*. *rhodopaea* (Velen.) Janch.), *Scabiosa triniifolia* and *Thymus longidentatus*. Eleven plants were included in the CITES Convention. A mound near the town of Radomir (ID 185) contained the richest number of plants of conservation interest and maintained populations of 5 such species.

**Figure 3:**
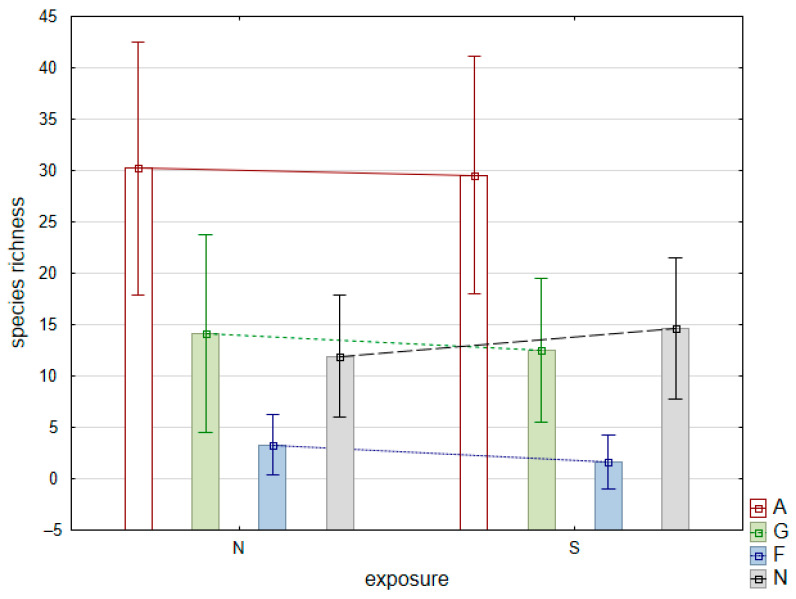
Differences in mean species richness (columns) and standard deviations (whiskers) of different species groups (A—all plant species, G—grassland specialists, F—forest specialists and N—generalists) between plots with northern (N) and plots with southern (S) exposure on the studied mounds.

According to our SIMPER analyses, species composition similarity across plots with northern exposure was 13.25% and was 11.84% across plots with southern exposure. We found a remarkable dissimilarity between plots with northern and southern exposure—88.95%. We registered 142 species unique to the plots with southern exposure. Species registered in more than three plots on southern slopes were *Camelina sativa*, *Heliotropium europaeum*, *Sedum hispanicum*, *Senecio leucanthemifolius*, *Valerianella dentata*, *Verbascum ovalifolium*, *V. densiflorum*, *Tribulus terrestris*, *Crepis sancta*, *Haplophyllum suaveolens*, *Herniaria incana*, *Thymelaea passerina* and *Filago arvensis*. Another 152 species were confined to plots with northern exposure. Taxa that exhibited higher frequency on the plots with northern exposure included *Astragalus glycyphylloides*, *Ficaria verna*, *Carlina vulgaris*, *Helleborus odorus*, *Leucanthemum vulgare*, *Phlomis tuberosa*, *Ranunculus polyanthemos*, *Anthoxanthum odoratum*, *Helictochloa compressa*, *Quercus frainetto* and *Luzula campestris*. The mean species richness of grassland and forest specialists and generalists was similar between plots with northern and plots with southern exposure (Figure 3).

Average dissimilarity between all pairs of mounds was 0.78 (Whittaker measure for beta diversity). Fifteen species were registered on more than 50% of the studied objects and included *Poa angustifolia* (77% of the studied mounds), *Achillea millefolium* aggr. (72.6%), *Eryngium campestre* (67.3%), *Galium verum* (64.6%), *Teucrium chamaedrys* (61.1%), *Tragopogon dubius* (60.2%), *Sanguisorba minor* (54%) and *Botriochloa ischaemum* (50.4%). According to our SIMPER analyses, these taxa along with *Prunus spinosa*, *Lactuca serriola*, *Dactylis glomerata*, *Falcaria vulgaris*, *Convolvulus arvensis*, *Crataegus monogyna*, *Elytrigia repens*, *Galium aparine* and *Potentilla recta* contributed the most to the floristic similarity between mounds. On the other hand, 480 species (45.3% of the total flora) were registered from one or two mounds. We found no relationship between the 2D area of the mounds and their floristic richness. With the enlargement of the 2D area of the mounds, the richness of forest specialists slightly increased (*r* = 0.28, *p* < 0.05). More than a half of the studied mounds (69) were highly isolated and surrounded by an agricultural matrix above the average in the buffer (Table 1). As the anthropogenically transformed lands around the mounds increased, the total number of registered species (*r* = −0.28, *p* < 0.05) as well as the total number of specialists (*r* = −0.34, *p* < 0.05), including grassland specialists (*r* = −0.33, *p* < 0.05), decreased.

**Figure 4:**
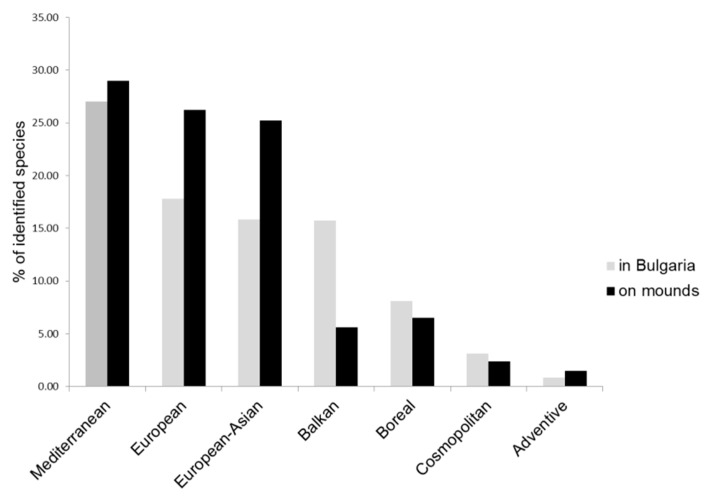
Phytogeographical spectrum of vascular plants on the mounds, compared to vascular plants in the Bulgarian flora. The national record was calculated by following [45].

The phytogeographical spectrum of the registered plants resembled that of the national flora (Figure 4). The number of species with Mediterranean distribution was the highest, followed by the plants with European and Eurasian distribution.

**Figure 5:**
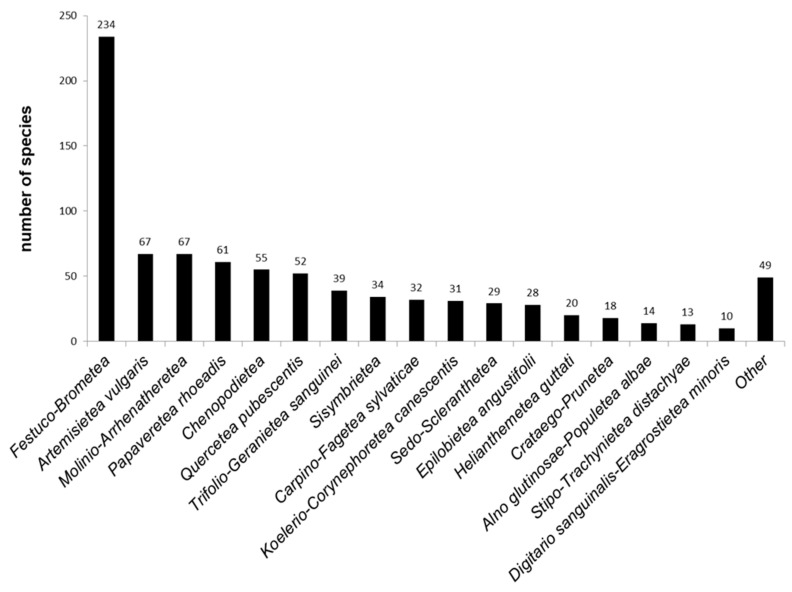
Vascular plants number associated with the phytosociological class level.

Across all plants growing on the mounds, 853 were diagnostic of 40 different vegetation classes. Most numerous were the grassland specialists and generalists, and *Festuco-Brometea* diagnostic species constituted 44% of all identified plants (Figure 5). There were 193 species diagnostic of anthropogenic vegetation (classes *Artemisietea vulgaris*, *Papaveretea rhoeadis*, *Chenopodietea*, *Epilobietea angustifolii*, *Polygono-Poetea annuae* and *Sisymbrietea*) and their per mound abundance was relatively low (on average from 2.2 ± 1.8 SD for *Chenopodietea* to 7.87 ± 3.5 SD species for *Artemisietea vulgaris*). There were 100 species diagnostic for forest vegetation (classes *Alno glutinosae-Populetea albae*, *Carpino-Fagetea sylvaticae*, *Quercetea pubescentis*, *Quercetea robori-petraeae* and *Salicetea purpureae*). Plants diagnostic for *Quercetea pubescentis* prevailed with the highest average per mound presence (2.56 ± 2.5 SD). There were 206 species with wide ecological plasticity to which no diagnostic value toward a particular syntaxon was attributed.

#### 3.1.2. Bryophytes

We registered a total of 58 bryophyte taxa; 54 were identified to species level (Supplementary ESM S1). They belong to 39 genera and 16 families. Pottiaceae included the highest number of species, all of which were confined to dry, skeletal and sandy substrates. The second most diverse family was Bryaceae, a group which includes species with diverse ecological preferences. On the mounds, this family was represented by species typical of dry eroded terrains. No bryophytes were registered on 40 of the studied mounds (36.04%). In cases where bryophytes were found, their number ranged from 1 to 10 species (average 3.1 ± 2.1SD). The only typical epiphytic moss recorded during this study was *Orthotrichum pumilum*, which was found on trees of the genus *Quercus*. The rest of the listed species usually occupied the soil substrate and were occasionally also found on woody stems. *Abietinella abietina*, *Barbula unguiculata*, *Pterygoneurum ovatum*, *Rhynchostegium megapolitanum* and *Thuidium assimile* were confined to herbaceous habitats. *Bryum pallescens* has broad ecological affiliation, while *Atrichum undulatum*, *Fissidens taxifolius* and *Plagiomnium affine* were observed only within the forested northern slopes. Nearly all of the bryophytes registered during the present study are taxa commonly found in Bulgaria where they are known to occur in a variety of different plant communities, usually in lowland areas. Phytogeographically, most of the bryophytes (35 species) belong to the temperate region. A significant share of the registered bryophytes were cosmopolites (*Polytrichum juniperinum*, *Polytrichum piliferum*, *Funaria hygrometrica*, *Schistidium apocarpum*, *Ceratodon purpureus*, *Weissia controversa*, *Syntrichia ruralis*, *Bryum argenteum*, *Pohlia nutans* and *Hypnum cupessiforme*). The *Ceratodon conicus* species, previously known from a single locality in Bulgaria (Vitosha Mt. [46]), deserves special attention. This taxon was registered on a mound in the Thracian lowland near the town of Plovdiv (ID249), a substantial distance from the previously reported location.

#### 3.1.3. Lichens

A total of 61 lichen taxa, 56 identified to species level, were registered on 52% of the studied mounds (Supplementary ESM S1). The highest number of recorded lichens were epiphytes (35 taxa), followed by epigean taxa (16 taxa), and by species with wide ecological breadth occurring on all substrate types (2 taxa). We recorded eight epilithic lichen taxa. The number of species registered on a mound ranged between 1 and 18 taxa (average 4.0 ± 2.1SD). Lichens were not registered on 57 of the studied mounds. The most common family found on mounds was Parmeliaceae, followed by Cladoniaceae and Physciaceae. Frequently registered lichen species were *Xanthoria parietina*, *Parmelia sulcata* and *Physcia adscendens*. *Arthopyrenia salicis* is recorded for the first time for the country. Until present, *Caloplaca cerinella* and *Catillaria nigroclavata* were known from single Bulgarian localities and are reported here for a second time. Phytogeographically, many of the lichens (31 taxa) are typical for the temperate region. In general, there is a clear preponderance of warm-temperate lichens. Arctic–alpine lichens, common for the Bulgarian alpine and subalpine zones, were not encountered during the present study. It is interesting to note that the new country record and the two other poorly recorded taxa were found together on a single mound (ID 562) situated in the eastern part of the country, near the Black Sea.

## 4. Discussion

### 4.1. Species Diversity

This article presents new information regarding the vascular and cryptogam (bryophytes and lichens) flora of ancient mounds in Bulgaria. The species diversity established during this study is a testament to the importance of ancient mounds for preserving the native flora as previously emphasized for other parts of Eurasia [3,12,13,15]. According to the most recent report [47], the Bulgarian flora includes 4064 vascular plant species. The 1056 vascular plant taxa we recorded present nearly a quarter of the overall national plant diversity. These results emphasize the role of ancient mounds in the preservation of a high percentage of the national floristic diversity. Similar results have been previously reported in other European countries: a total of 346 plants were registered on 82 mounds in Hungary [18], which equals 15.5% of the national floristic diversity [48] and 721 species were registered on 106 mounds in Ukraine [12], which represents 11.6% of the national flora [49]. The flora of the studied ancient mounds mirrors the phytogeographical characteristics of the Bulgarian flora, i.e., the mounds contain a representative sample of the national plant species pool. The higher proportion of Mediterranean and European, as well as adventive, elements reflects the species pool of the lowlands. A similar finding, namely that ancient mounds reflect local biogeographical zones, was reported by Sudnik-Wójcikowska and Moysiyenko [50], in Ukraine.

We expected that the flora of the mounds in different countries will reflect local environmental conditions, species pools and human influence. Nevertheless, there was a surprising similarity regarding the number of species recorded per mound in different countries. Our results were close to the 72 species registered on the best documented mound, Csípő-halom (Hortobágy, Great Hungarian Plain) [51]. The range of species established per mound in Poland has been found to be from 44 to 81 [52], and the reported number of species per mound in Ukraine has been found to be between 82 and 125 [12]. Similarities among the mounds in Europe did not solely concern their species diversity. Another similarity is the weak correlation between mound area and species richness, originally established by Deák et al. [53]. Our results corroborate these findings and strengthen the idea that mound protection should be of high priority irrespective of their size. Studies of ancient mounds often refer to these structures as islands in a sea of anthropogenically modified areas [12,14,15]. This is a fair comparison, given the fact that the increase in agricultural and other modified areas around the mounds does not significantly affect their biodiversity, a finding also confirmed by our study.

The outstanding floristic diversity of Bulgarian mounds, especially given the fact that most of them are located in areas characterized by intensive large-scale agriculture, supports our assumption that ancient mounds are valuable refugia for indigenous flora and that they play an important role in its conservation. The number of species we registered on one or two mounds was high (45.3% of the established flora) and it reflected the local species pool, a finding similar to that of Sudnik-Wójcikowska and Moysiyenko [8]. The high rate of dissimilarity between the sampled mounds is a reflection of the diversity of biogeographical zones in the country. The difference in species composition between plots with northern and those with southern exposure corresponds to the well-established effect of slope aspect on vegetation [54]. The abundance of generalists on the south facing slopes and the abundance of grassland specialists on the northern slopes support the findings of Deák et al. [9]. Forest specialists are more abundant on north facing slopes due to the ecological requirements of trees to milder climatic conditions and especially to higher air and soil moisture.

According to our results, 6.4% of the bryophytes native to the country can be found on the mounds. The new *Ceratodon conicus* locality, established nearly a century later and on a substantial distance away from the original locality, gives us a reason to believe in the potential of ancient mounds for the long-term preservation of biodiversity. The cover and diversity of lichen species depends primarily on suitable substrates and environmental factors. Although the saxicolous lichens represent one of the largest ecological groups of lichens, their preferred bare and stable rock substrates were not found on the studied mounds. Most of the saxicolous lichens found during this study appeared on artificial substrates of contemporary anthropogenic structures, such as geodetic points. Only a few species were found on pebbles that are not a suitable substrate for lichens because of their instability. The predominance of epiphytic lichens is not surprising as most of the mounds had trees and shrubs. The terricolous taxa were rarely present due to their inability to compete with the well-established vegetation. The first record of the epiphytic lichen *Arthopyrenia salicis* from Bulgaria is noteworthy. To our knowledge, the closest *A. salicis* locality is in the Slovenian Julian Alps [55]. This species is especially frequent in northern and western Europe [56], where it occurs on the smooth bark of the trees, as experienced in our study. Lichens frequently established on the mounds are among the most common species characteristic of the lowland epiphytic communities on broadleaved trees in Bulgaria. However, the abundance of *Xanthoria parietina* and *Physcia adscendens* is indicative of a higher load of nitrogenous compounds [57]. Typical nitrophilous lichen community members, such as *Phaeophyscia orbicularis* and *Polycauliona polycarpa* [58], were also relatively frequently found on the studied mounds. We assume that these nitrophilous species might have been positively affected by the surrounding agricultural lands and treated with fertilizers.

### 4.2. Species Characteristics

The ecological characteristics of the established flowering plants strongly resemble the peculiarities of the national flora with a dominant presence of species typical for dry areas. These species use evolutionary advantages to survive via vegetative propagules (e.g., *Agropyron repens*, *Poa angustifolia*, *Botryochloa ischaemum*, *Achillea millefolium*, *Bryum dichotomum*, *B. klinggraeffii*, *B. moravicum*, *B. rubens* and *B. ruderale*), underground storage organs (e.g., *Arum maculatum*, *Crocus flavus* and *Ornithogalum* spp.) or via annual life cycles (e.g., *Trifolium striatum*, *Astragalus spruneri*, *Apera spica-venti*, *Arenaria serpyllifolia* and *Coronilla scorpioides*). Patches of bare ground and pits left after continuous illegal treasure hunting become suitable places where fruits or seeds of common trees and shrubs (*Pyrus pyraster*, *Prunus cerasifera*, *P. spinosa*) accidentally fall and develop free from competition. Open spaces also favor the establishment and continued persistence of certain bryophytes confined to eroded habitats (*Ceratodon purpureus*, *Polytrichim piliferum* and *Bryum argenteum*).

The role of ancient mounds in steppe preservation is continuously being reiterated in the published literature [3,6,7,17,59]. Our study confirms the prevalence of species characteristic of the *Festuco-Brometea* class associated with the mounds, similar to those reported for Ukraine [6]. This vegetation type includes dry grasslands and steppes from the sub-Mediterranean, nemoral and hemiboreal areas of Europe [30]. *Festuco-Brometea* is widespread in the lowlands and hilly plains of Bulgaria and unites mainly secondary herbaceous communities. Some exceptions occur in the north-eastern parts of the country where small fragments of true steppe communities are present. Given the anthropogenic origin of the mounds, a large number of anthropophytes are logically expected to be associated with these structures. We identified 19.9% of all species as related to anthropogenic vegetation of the classes *Artemisietea vulgaris*, *Papaveretea rhoeadis* and *Sisymbrietea*. Species characteristic of *Artemisietea vulgaris* provide a signal for ruderalization associated with dry habitats. Species diagnostic of *Papaveretea rhoeadis* and *Sisymbrietea* reveal the apparent influence of segetal vegetation in the mounds’ immediate vicinity. The diagnostic features of the plant taxa registered on the mounds gives us a reason to characterize most of their vegetation as ruderalized steppes. Although the ancient mounds are considered to be important areas for the protection of steppe flora and vegetation [3], in Bulgaria they also preserve fragments of forest communities [4] characteristic of temperate lowlands. Most of the established trees and shrubs belong to *Quercetea pubescentis* and *Carpino-Fagetea sylvaticae*, two classes widely distributed on the territory of the country. The development of trees, which were visibly old in some places, is a result of the natural succession directed toward the potential vegetation of the Bulgarian lowlands under temperate climate [21]. This successional trend is also indicative of the apparent lack of management practices and activities as associated with the ancient mounds. The diversity of tree species and forest specialists was low compared to the overall floristic diversity. However, the increasing tree coverage leads to a decreased biodiversity under the canopy [60].

### 4.3. Significance for Nature Conservation

Preserved for 2–3 millennia, ancient mounds retain the natural flora as evidenced by the predominant presence of native species associated with these structures. The prevalence of perennials is a testament to the long-lasting stability of the local vegetation and creates conditions for the establishment of cryptogams. The lack of serious disturbances over prolonged periods, excluding archaeological investigations and damaging activities by treasure hunters, has favored the establishment of perennial species, respective of closed vegetation, which prevents the penetration of many neophytes and especially that of invasive alien plants. The common development of communities in vascular plants and cryptogams is considered an indication of sustainability [61].

Vascular plants of conservation importance were not common on the ancient mounds. The observed endemic species presented only 5.2% of the Balkan endemics reported for Bulgaria [36]. The sampled mounds preserve two critically endangered and nine endangered plants, which represent 1.8% of both categories evaluated at the national level [37].

Our results corroborate the notion that the undisturbed closed vegetation prevents establishment of alien invasive plants [62]. The presence of low neophytes and alien plant numbers has been previously reported for other archaeological sites [63]. Nevertheless, our results hint towards an impending threat given the proportion of mounds affected by the presence of even sole individuals of alien plant species. Although the presence of large agricultural fields isolates the mounds from immediate human influence, we found no evident relationship between the surrounding land use and the number of anthropophytes present on the mounds. Similar results were previously reported by Sudnik-Wójcikowska and Moysiyenko [64].

The existence of relatively well-preserved floristic diversity indicates that the mounds have the potential to provide not only cultural and spiritual but also other valuable ecosystem services (e.g., provisioning of biomass, maintenance of native plant populations and maintenance of physical, chemical and biological conditions of the locality) [65], which, in the face of accelerated fragmentation and land degradation, will become even more important in the future. Therefore, there is a clear need for further research on the topic. The high rate of isolation on the mounds along with their floristic richness, emphasize their remarkable role in the preservation of natural communities, and in providing connectivity when serving as stepping stones for species dispersal from and to other fragments of natural and semi-natural environments, as previously mentioned by others [7].

The Natura 2000 and the network of protected areas are naturally considered as part of the EU’s strategy for green and blue infrastructure, but new areas are expected to be added in the future [10]. Within the growing body of literature regarding the concept of green infrastructure, we perceive green infrastructure as green space planning [66]. Situated in anthropogenically transformed lands, the mounds present ideal features for greenspace preservation. Some of Bulgaria’s ancient mounds are situated within urban areas, but we did not include them in our sampling. The presence of semi-natural (green) space within arable fields will certainly support an increase in environmental benefits. Ancient mounds are existing structures that only need maintenance and protection. Protection from further treasure hunting is required because such disturbances could facilitate the penetration of alien and woody plants. Reduction of shrubs (especially *Prunus spinosa*) and non-native trees (*Robinia pseudoacacia*, *Ailanthus altissima*) will help to maintain species-rich grasslands. Although species of conservation importance were rarely observed during our study, the mounds should not be neglected as important areas for plant protection, especially in the cases of highly fragmented semi-natural areas where different subpopulations are well separated from each other.

## 5. Conclusions

Millennia-old ancient mounds are a characteristic feature of the Bulgarian landscape and play an important role for the conservation of indigenous flora. Agricultural practices in the surrounding areas have little effect on the floristic richness of the mounds. In this study, we show that Bulgaria’s ancient mounds preserve a considerable proportion of the national vascular and cryptogam flora. Further research will certainly offer new knowledge about the natural significance of the ancient mounds. Current legal preservation of the ancient mounds as archaeological monuments does not guarantee their proper management in terms of nature conservation. With the enlargement of the green infrastructure in the cultural landscape at European level, the significance of ancient mounds will most certainly increase.

## Figures and Tables

**Table 1:** Basic topographic parameters of the studied mounds (n = 111).

Parameter	Min	Max	Mean	SD
Altitude (m, a.s.l.)	61.0	920.0	354.4	238.8
Height (m)	1.2	24.5	5.3	3.1
Diameter (m)	9.5	88.4	40.7	15.8
Coverage of herbaceous vegetation (%)	0	100	78.3	30.5
Coverage of shrub/forest vegetation (%)	0	100	21.7	30.5
Surrounding of semi-natural vegetation in 200 m buffer (%)	0	100	23.1	26.4
Surrounding of agricultural and other anthropogenic lands in 200 m buffer (%)	0	100	76.9	26.4

**Table 2:** Biological characteristics of the registered vascular plants. Total number and descriptive statistics are given.

	On a Mound				
Parameter	Total	Min	Max	Mean	SD
Biological type					
Short-lived	378	4	64	24.77	10.90
Perennial	495	10	88	36.13	13.57
Dwarf-shrub	17	0	7	0.80	1.09
Shrub	49	0	12	4.02	2.80
Tree	35	0	7	2.55	1.87
N/A	85	0	10	2.20	2.26
**Plants with conservation importance**					
Critically Endangered (CR)	2	0	1	0.04	0.19
Endangered (EN)	9	0	2	0.22	0.43
Vulnerable (VU)	7	0	2	0.15	0.39
Near Threatened (NT)	6	0	1	0.07	0.26
Least concern (LC)	2	0	2	0.21	0.45
Bulgarian Biodiversity Act	10				
Habitat Directive	1				
CITES	11				
N/A	85	0	10	2.20	2.26
No conservation status	947	27	137	67.50	20.87
**Native status**					
Native (including archaeophytes)	948	27	141	66.76	21.30
Naturalized alien	5	0	2	0.15	0.39
Doubtfully native	1				
In large-scale cultivation	2				
Alien (status unknown)	13	0	5	0.83	0.97
N/A	85	0	10	2.20	2.26
No data	5	0	2	0.45	0.63
**Invasive alien plants (for Bulgaria)**					
Invasive alien plants (IAP)	12	0	4	0.50	0.80
Not classified as IAP	962	27	142	67.76	21.38
N/A	85	0	10	2.20	2.26

## Data Availability

All data analyzed in this study are available in Supplementary ESM S1.

## Supplementary Materials

The following supporting information can be downloaded at: https://www.mdpi.com/article/10.3390/plants11050705/s1. ESM S1: Original floristic data from 111 ancient mounds from Bulgaria.
